# Developing a consensus statement for psychosocial support in active surveillance for prostate cancer

**DOI:** 10.1002/bco2.155

**Published:** 2022-05-06

**Authors:** Kerri Beckmann, Declan Cahill, Christian Brown, Mieke Van Hemelrijck, Netty Kinsella

**Affiliations:** ^1^ Translational Oncology and Urology Research Kings College London London UK; ^2^ Cancer Epidemiology and Population Health Research Group University of South Australia Adelaide South Australia Australia; ^3^ Department of Urology The Royal Marsden Hospital London UK; ^4^ Urology Department Guy's and St Thomas' NHS Foundation Trust London UK

**Keywords:** active surveillance, consensus statement, Delphi study, prostatic neoplasms, psychosocial support systems

## Abstract

**Purpose:**

Our objective was to prioritise the psychosocial support needs of men on active surveillance for prostate cancer and to develop a consensus statement to provide guidance on best practice psychosocial support for men choosing active surveillance and their families.

**Subjects and methods:**

We undertook a patient and public involvement Delphi process over two rounds, informed by qualitative data and a comprehensive literature review, to prioritise the information and support needs of men on active surveillance for prostate cancer. Two panels were surveyed, a patient/carer panel (*n* = 55) and a health care provider panel (*n* = 114). Based on the findings of the Delphi surveys, an expert active surveillance discussion group developed a consensus statement to guide best practice.

**Results:**

Patients and health care professionals differed slightly in their ideas concerning priorities for active surveillance psychosocial support. Broadly, agreed priority areas included ‐patients being involved in decision‐making, continuity of care, more streamlined access to health care teams, improved understanding of the risk of prostate cancer progression and information and support provided through both health care professionals and peers. Based on the identified priorities, the expert discussion group agreed on 22 consensus statements for best practice in psychosocial care for active surveillance in respect of (1) principles of an active surveillance programme; (2) structure of consultations; (3) content of information and support; and (4) delivery of information.

**Conclusion:**

This consensus statement provides a framework for patient‐focused psychosocial support, which, if adopted, should increase uptake and adherence to active surveillance among men with prostate cancer.

## 1 INTRODUCTION

European guidelines suggest a large proportion of men with low risk prostate cancer (PCa) do not require immediate treatment but can be monitored—an approach known as active surveillance (AS).^1^ However, despite the obvious lack of morbidity associated with a surveillance programme versus radical treatment, AS drop‐out rates (up to 38%) remain high in men with no evidence of disease progression.^2^

Several studies have shown that supportive and informational interventions aimed at men on AS have a favourable impact on adherence. Oliffe et al.^3^ found that self‐management strategies helped men to cope with the long‐term uncertainty of AS, whereas the Prostate Cancer Lifestyle trial, which included exercise and attention to stress management, demonstrated an improvement in treatment‐free survival on AS.^4^ Goh et al.^5^ found that men who felt they were receiving useful and consistent information were more satisfied and therefore more likely to continue on AS, while the UK‐based ProtecT trial^6^ found merit in consistency of personnel to support and inform patients. Despite this, the provision of extra information and psychological support for men on AS continues to be consistently highlighted as an area needing improvement.^7^, ^8^

The objective of this project was to agree on the priorities for psychosocial support and to translate these into a useable consensus statement to inform the development of future interventions to support men and their partners/families undergoing AS for PCa.

## 2 PATIENTS AND METHODS

### 2.1 Establishment of an expert reference group

An expert active surveillance discussion group (ASDG) was established to guide all aspects of this study from start to end.^9^ This reference group included 13 participants: four men on AS, two men who had opted out of AS without evidence of progression, two partners, one representative from the local Patient and Public Involvement group, two health care professionals (HCPs, a Urologist and a specialist nurse) and two representatives from prostate charities. Members did not receive any reimbursement.

**FIGURE 1:**
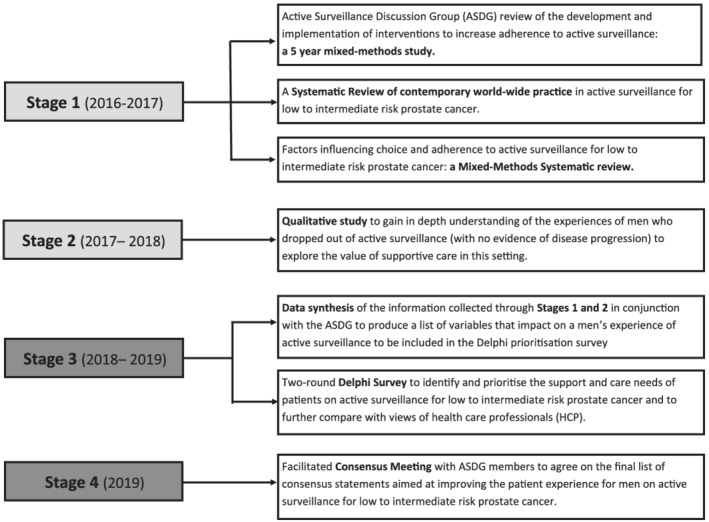
Schema presenting the phases of work undertaken to develop this consensus statement

Figure 1 shows the flow and timing of the programme of work undertaken to arrive at this consensus statement. This paper describes the third and fourth stages, which includes the synthesis of evidence, the Delphi process and consensus. Stages 1 and 2 were reported in detail in previous publications.^2^, ^10^, ^11^ The ASDG provided guidance on the overall design and gave feedback throughout all stages of the project.

### 2.2 Synthesis of evidence

To inform the development of the statement of support needs for men on AS, we conducted a syntheses of results from our previous qualitative interviews with men who discontinued AS^10^ and our mixed‐methods systematic review.^11^ We adopted the approach described by Voils et al.^12^ to aggregate findings from both studies to develop summative statements to inform the Delphi process that followed. The ASDG reviewed all potential statements prior to inclusion in the Delphi surveys.

### 2.3 Delphi prioritisation survey

Two Delphi survey panels were established, one targeting patient and carers and the other targeting HCPs. Separate surveys were written for each group, with appropriate language to describe the same items (Figures S1 and S2). HCPs were recruited via social media, with the assistance of the British Association of Urological Surgeons, British Association of Urology Nurses, British Uro‐Oncology Group and Specialist Urology Registrar Group. To qualify as Delphi participants, HCPs needed to have provided care to men on AS for low‐intermediate PCa in the past year. Patients and carers were recruited through The Royal Marsden, Epsom and St Helier and King's College Hospital Urology clinics. According to Wilkin and Altschuld,^13^ 50 participants is an adequate number to provide representative results from heterogeneous Delphi panels, which was reached for both panels.

We used an accepted modification of the Delphi process^14^ in which panellists received a structured questionnaire based on synthesis of our previous literature review and qualitative study findings, with a subsequent round to consolidate rankings of statements' importance. Both rounds were conducted within an 8‐week period to ensure continued engagement by participants.^15^ The first survey was live for 2 weeks, followed by a 2‐week analysis period. Round 2, conducted 10 weeks later, was available for 4 weeks. The survey was presented to participants in a block‐randomisation format to avoid the influence of respondent fatigue.^16^

Participants were asked to score each item on a scale from 1 (*not important*) to 7 (*most important*). The first round allowed participants to add further items as free text. First round results were reviewed and fed back to participants before the second round commenced. Items scoring ≥6 by >60% of participants in each round were selected for the consensus meeting, as advised by Belton et al. when the range of responses is varied.^17^

Survey completion was anonymous; however, demographic information was collected on age, level of education, relationship to patient, number of months on AS, area of practice and current profession (where appropriate). Questionnaires were administered through the General Data Protection Regulation (2018) compliant smart survey website.^18^

### 2.4 Consensus meeting

Following the Delphi process, members of the ASDG were invited to participate in a consensus meeting to agree on the priorities for support needs and translate these into a consensus statement to guide future development of interventions to increase participation and adherence in AS for PCa. This meeting was based on the nominal group technique,^19^ which is a highly structured face‐to‐face group interaction to empower participants to share their opinions, with moderation by an independent expert in consensus methods. Ulschak's^20^ recommendation that 80% agreement be reached among members for consensus was amended to 70% agreement because only a few items achieved the higher level. This likely reflects the diverse experience and perspectives of members of the ASDG, including those of patients, carers, charity representatives and health care professionals. This level of consensus was considered appropriate in other Delphi studies.^21^

### 2.5 Ethical approval

This study was approved by the Quality Improvement Project Committee at The Royal Marsden NHS Trust as part of a wider project implementing a new information and supportive care programme for men on AS. Consent was implied by participants' willingness to respond to the Delphi surveys. This work was undertaken as part of Doctorate thesis by the last author NK, who gives her permission for its publication in order to reach the broader Urology community.

## 3 RESULTS

### 3.1 Evidence synthesis

Our previous systematic mixed‐methods literature review^11^ identified both patient‐related and clinician/health system‐related factors that influence uptake and adherence to AS. Patient‐related factors included perceived risk of cancer, fear of progression, decisional conflict and lack of understanding or acceptance of AS. Clinician and health system factors included acceptance of AS as a valid management option, clinician recommendations, shared decision‐making and building of trust, the provision of information and support, variations in policies and practices across regions and lack of consist guidelines.^11^

Results from our qualitative interviews^10^ identified additional factors that impact men's sense of well‐being, leading to a reluctance to continue on AS. Key themes included negative experiences at diagnosis, continuing delays and anxieties relating to follow‐up appointments, lack of information and structured support, poor communication from the care team, exclusion from shared decision‐making and inflexibility within the system. A lack of information and support for family members and limited access to peers with similar experiences were also identified as potential barriers to remaining on AS.

Table 1 shows the synthesis of findings from both the qualitative interviews and our systematic review, grouped into themes related to cancer characteristics, patient factors, family and social support, provider issues, health care organisation and practice and health policy factors. Upon review by the ASDG, agreement was reached on the inclusion of 62 potential statements for inclusion in Delphi surveys, based on our synthesis of evidence. (See Figures S1 and S2.)

**TABLE 1:** Themes identified via meta‐aggregation and synthesis of data from qualitative interviews and mixed‐methods systematic review

Themes	Factors	Potentially targetable interventions for prioritisation
1. Cancer characteristics	Cancer risk, stage, grade, PSA, tumour volume	Harmonising national/local guidelines; developing consensus‐based appropriateness criteria. Improving the experience of diagnostic pathway—waiting time, type of appointment, Multidisciplinary Team recommendation
2. Patient	Shared or collaborative role in decision‐making; preferences; seeking information; feeling informed; knowledge	Shared decision‐making; appropriate, reliable and unbiased information; personal information; not contradictory and not stressful; increased availability of educational resources from trusted medical organisations for patients and families
	Patient characteristics (age, comorbidities, race, family history of prostate cancer, education, employment, insurance, socio‐economic status)	Physician judgement and recommendation in shared decision‐making with patient preference.
	Impact of long‐term AS on: (urinary function, sexual function); preservation of health‐related quality of life; time to accept diagnosis and to decide; ‘buying time’	Patient education and information, ‘prostate’ health reviews at consultations
	Self‐management support; preference‐style for diet, exercise and complimentary therapies; increased awareness and control of health; hope for prolonged and improved health; symptom monitoring; lifestyle	Patient education and information; self‐management through diet and exercise, stress and anxiety management, digital technology.
	Preference for immediate cure; ‘cut it out’; desiring treatment efficacy/cure; avoid future regret	Patient education and information; supportive counselling
	Perceived cancer risk; cancer worry; fear of disease progression; illness uncertainty; anxiety and distress	Patient education and information; support; coping; manage anxiety; cognitive reframing; mindfulness; meditation; empowering; support groups; peer community; socialisation; connect to others; shared activities; a sense of belonging; provide patients with a sense of meaning and control, robust monitoring processes, widespread agreement on monitoring process.
	Patient education and information; support; coping	Patient education and information; support; coping
	Awareness and acceptance of AS; survival expectation on AS	Availability of AS ‘success stories’
	Unknown factors	Qualitative interview studies with physicians and patients
3. Family and social support	Advice/pressure from partner/spouse/children/friends; marital status; family member with PC	Supportive counselling and information; patient not having to justify decision to others; support; education; reassurance
	Awareness and acceptance of AS	Public role models managed with AS and patient advocates
	Fear of progression; disagreement about safety; preference to ‘eradicate the cancer’	Counselling and information; enhanced recognition with information sources, treatment support, multidisciplinary consultations (doctor and nurse), expert patients, peer group meetings (support)
4. Provider	Physician's recommendation; consistency in medical/nursing personnel	Training specialists to use a systematic approach to counselling patients about treatment options; communicating clearly and with confidence; using nudging narratives and framing techniques from behavioural science theory; maintain a positive and hopeful attitude; provide support and reassurance; public reporting of physicians' cancer management profiles
	Specialty of physician giving treatment information	Multidisciplinary team of specialists
	Provision of information and support	Provide and direct patients to accurate and unbiased information rather than describing AS as ‘doing nothing’ or ‘no treatment’ or scaring patients to active treatment, access to AS support groups. Establish consistency of support through nurse specialist roles, type of education offered, expert patients
	Physician attitudes; reluctance; concern about disease progression; perceived lack of data	Raise awareness, on‐going discussions at national meetings, quality improvement initiatives; having clear plans and stopping rules; systematic counselling on AS
	Lack of availability of physicians recommending AS	Advocacy; subspecialty within urology
	Confidence and trust in health professionals; closeness with physician; share control over treatment decision‐making	Improved community and medical education about treatment options, prognosis, side‐effects; raise awareness of AS; consistent, unbiased treatment information; decisional support information; building trust in physician; patient trusting physician's monitoring; patient feeling AS is an organised, supportive process
	Administration of surveillance pathway	Waiting times, administrative access, appointment type, web‐based organisation of bookings and cancellations, biopsy and imaging management and results review
5. Health care organisation/practice	Urology practice site; hospital referral region; geographic region	Quality improvement initiatives to harmonise practice sites within networks
	Degree to which physician shared control over treatment decision‐making	System‐level determinants of trust, closeness and shared decision‐making; organisational changes, e.g., longer consultation times
	Consultation at a multidisciplinary clinic; university hospital setting; academic hospital or high volume of PC patients	Multidisciplinary clinic may reduce the bias that specialists prefer the modality of treatment they themselves deliver and patients receive a balanced perspective of risks and benefits of options
	Differences in surveillance strategies	National/International consensus of safe AS. Selection, monitoring and progression, patient information on large AS cohorts
6. Health policy level	Guideline recommendations	Harmonising national/local guidelines; developing appropriateness criteria; national guideline recommending AS; real‐time feedback to units on adherence to national guideline in terms of annual report publicly available online
	Trial/cohort data; year of diagnosis	Monitoring and future publications from on‐going, prospective protocol‐based AS cohorts and registries
	Awareness and acceptance	Guidelines; consensus; discussions at meetings; AS‐specific billing code

Abbreviations: AS, active surveillance; PC, prostate cancer.

### 3.2 Delphi surveys

Fifty‐five people took part in the patient/carer surveys. Participants included 36 men currently on AS, 16 partners/family of men on AS and 3 men that were previously on AS. The median time on AS was 24 months (range: 6–156 months), 73% of participants were ≥60 years of age, 71% had at least a university diploma or degree and most were of White British heritage (Table S1). A further 114 individuals took part in the HCP surveys; 71% were ≤49 years of age, 79% were doctors and 52% were clinically active in South England, whereas 13% practised outside of the United Kingdom (Table S2).

Table 2 compares the level of agreement between patient/carer and HCP survey responses by identifying all items according to the percentage of participants who scored the item as ≥6 (6 = *much more important*, 7 = *most important*).

**TABLE 2:** Prioritisation of factors to improve psychosocial support in active surveillance for prostate cancer, top ranking items

Priority areas		% Patient and carer panel scoring ≥ 6	% Health care professional panel scoring ≥ 6
Patient factors			
1	Patients feeling involved in decisions about active surveillance, e.g., scans and re‐biopsy	89	90
2	Access to reliable sources reporting on the latest research in prostate cancer and active surveillance	84	59
3	Good mental health while on active surveillance	82	77
4	Good physical health while on active surveillance	81	80
5	Recommendation from the hospital clinical team	79	85
6	Partners/family awareness and knowledge of active surveillance	72	79
7	Including partner/family in consultations and cancer decisions	70	82
8	Partners/family acceptance of active surveillance	67	79
Cancer factors			
1	Understanding the risk of prostate cancer progressing	81	94
2	Understanding other treatment options for low‐risk prostate cancer	72	90
3	Understanding prostate cancer	70	91
4	Control of health—including regular assessment of any prostate related symptoms, e.g., urinary symptoms	70	73
5	Understanding the pathology of prostate cancer (Gleason grade)	65	72
6	Understanding the role of PSA in active surveillance	67	83
7	Understanding MRI scans and the role they play in active surveillance	61	74
Health care provider factors			
1	Consistently seeing the same clinical team (doctor or nurse)	79	83
2	Access to reliable information about active surveillance	74	91
3	Sharing treatment decision‐making with the clinical team	72	88
4	Easy access to the clinical team	67	87
5	The clinical team supporting and recommending active surveillance	63	92
6	Regular contact with the clinical team (nurse or doctor) via phone	44	77
Health care organisation factors			
1	Agreement on guidelines (Clear National or Local guidelines) for safe active surveillance	79	78
2	Reduce difficulty contacting the clinical team	79	82
3	Being monitored by a team/clinician with a special interest in active surveillance	77	75
4	Reduce difficulty contacting the administrative team	39	75
Support and Information content and delivery			
1	Education delivered by a health care professional (doctor, nurse, physio, etc.)	77	81
2	Exercise advice	71	43
3	Lifestyle advice	72	49
4	Public role model stories	70	59
5	Face to face ‘information and support seminar’ given to a group of men on active surveillance (hospital base)	31	31
6	Face to face ‘information and support seminar’ given to a group of men on active surveillance (community space)	26	18
7	Online website (webinar)	22	12
8	Printed information	21	39
Follow‐up method			
1	Face to face appointments	82	67
2	Offered my preferred method of contact for follow‐up	81	59
3	Follow‐up by a hospital doctor	60	71
4	Seen in a specialist active surveillance clinic	58	78
5	Follow up by a specialist nurse	47	87

*Note*: Items are ordered in accordance with the priorities of the patient/carer group.

Abbreviation: PSA, prostate specific antigen; MRI, magnetic resonance imaging.

While there was general agreement on important patient‐related factors, the second highest priority identified by patients and carers was access to reliable sources reporting on the latest research in AS, which in contrast did not appear at all on the HCP priority list. In addition, HCPs ranked partner/family acceptance of AS within five most important statements AS (79%), whereas the patient/carer group rated this issue more moderately (67%).

The top three cancer‐related factors identified by the patient/carer group were also recognised by HCPs. The HCP group felt that an understanding of Gleason grade (72%), prostate specific antigen (PSA) levels (83%) and magnetic resonance imaging (MRI) (74%) was of importance, whereas the patient/carer group found these items less important, with 65%, 67% and 61% of participants, respectively, rating these factors highly.

There was also general agreement across items relating to organisational factors, although HCPs felt that administrative issues may significantly influence AS adherence (75%). This was not reflected in patient/carer responses, where this was rated of low significance (39%). However, when asked specifically about the cancellation of appointments, scans or biopsies, both stakeholder groups viewed this as an important barrier to AS adherence (70% of the patient/carer group and 71% of the HCP group).

In reference to support and information required, there was agreement between patient/carer and HCP respondents regarding the importance of general education delivered by a HCP. Although the patient/carer group agreed on the importance of exercise and lifestyle advice, and hearing about the AS experience from public role models, they did not reach consensus regarding the method of delivery of information and support, with equal weighting given to webinars, seminars and printed information.

In reference to follow‐up method, there was no specific agreement between patient/carer and HCP groups. The patient/carer group felt that the only important aspect of follow‐up was being seen face to face, whereas the HCP group was more concerned that patients received consultation from a hospital doctor, nurse specialist and/or were seen in a specialist AS clinic. Patient/carer agreement on prioritising these three factors was moderate to low, with 60%, 47% and 58% agreement of importance, respectively.

### 3.3 Consensus statement development

From the Delphi surveys, 28 statements that achieved scores ≥6 by ≥ 60% of participants were identified. Members of the ASDG then ranked these statements from most to least important and provide their respective reasoning.^22^ Following mediated discussions, 22 consensus statements were formulated from these results. These statements were allocated across four themes: (1) Principles of an active surveillance programme; (2) Structure of consultations; (3) Content: information and support; (4) Delivery of information (Table 3).

**TABLE 3:** Consensus statement for active surveillance information and support needs

Consensus statement	
Principles of an active surveillance programme	
1	Ensure all patients have easy access to the clinical team (nurse or doctor)
2	Develop nationally agreed guidelines for safe active surveillance
3	Ensure health care professionals routinely assess mental health and provide access to appropriate psycho‐social support for men on active surveillance
4	Ensure health care professionals provide lifestyle advice to men on active surveillance, e.g., smoking cessation, reducing alcohol and eating healthily
5	Ensure health care professionals are educated about and actively promote exercise interventions to men on active surveillance
Structure of consultations	
1	Offer the appointment type that best suits the patient, e.g., telephone, face to face and skype
2	Offer patients their preferred contact method for appointments and outcome of consultations (email, text, letter, phone)
3	Ensure patients are consistently seen by the same clinical team (doctor or nurse)
4	Include regular assessment of prostate‐related symptoms, e.g., urinary symptoms, erectile function
5	Offer active surveillance monitoring by a clinical team with a special interest in active surveillance
Content: information and support	
1	Ensure patients are actively involved in decisions about active surveillance, e.g., repeat scans and re‐biopsy
2	Encourage the inclusion of partner/family in education and consultations
3	Ensure access or signposting to structured peer support locally
4	Ensure patients understand the risk of prostate cancer progressing
5	Ensure patients understand the other treatment options for prostate cancer
6	Provide access to up‐to‐date research on large active surveillance studies
7	Ensure patients understand the role of PSA/Gleason grade/tumour volume in active surveillance
Delivery of information	
1	Offer face to face ‘information and support seminar’ given to a group of men on active surveillance (hospital based)
2	Offer face to face ‘information and support seminar’ given to a group of men on active surveillance (at a local community centre)
3	Signpost to reliable online websites/webinars
4	Offer/signpost (unbiased) to paper based information resources (from charities)
5	Where possible actively signpost patients to stories about public role models on active surveillance

## 4 DISCUSSION

There are no currently available national or international guidelines pertaining to psychosocial support in AS. Our Delphi process identified 22 priority statements for successful psychosocial support. These encompass promoting good physical and mental health while on AS by providing professional exercise and lifestyle advice and ensuring easy access to clinical teams; offering more flexibility in the types of consultation and contact methods while ensuring continuity of care by clinical teams with specialist interest in AS; encouraging shared decision‐making and including partners and family; increasing patients knowledge of PCa, treatment options and risk of progression; providing broader lifestyle advice; and finally, offering face‐to face information and support seminars that are either hospital based or community based as well as providing signposts to unbiased online resources and public role models. In addition, as highlighted by Merriel et al. in a recent international Delphi study,^23^ agreement of appropriate selection criteria for AS and development and adoption of agreed guidelines for the safe delivery of AS will provide greater reassurance and potentially greater long‐term adherence, with obvious merits in reducing the morbidity and health care burden of overtreatment.^24^

Through undertaking a systematic review of the literature, semi‐structured interviews and an extensive Delphi survey involving patient and HCP perspectives, we have developed statements that reflect patient needs and provide achievable guidance within the health care setting. The variation in priorities given to some aspects of care by the patient/carer and HCP panels highlights the importance of implementing a robust patient and public consultation process when designing future interventions aimed at increasing AS adherence.

A more patient‐centred approach to AS, as outlined in our consensus statement, is required to address men's information and support needs. Organisations such as National Health Service Improvement (an independent charity committed to better health and health care) and the Heart Foundation have proposed strategies for supporting self‐management to improve health outcomes, based on the premise that if people are educated about their condition and feel confident of its management, promoting self‐management will ultimately increase adherence to chosen treatments.^25^ These include the development of ‘stratified’ models of care and support that take account of the needs of the patient in relation to the treatment received to achieve a more personalised approach leading to a greater degree of satisfaction.^26^ Such ‘person‐centred’ approaches have been shown to create a more positive experience of care leading to increased satisfaction; lead to the adoption of healthier lifestyles, for example, exercise and diet; encourage involvement in decisions about their care and ensure services and support is appropriate for their needs; improve health outcomes, for example, weight and fitness; and increase adherence to treatment.^27^

Health care teams, however, have been slow to adopt a more personalised supportive care approach, partly due to the perceived investment requirements. However, recent guidance on implementation suggests that adopting follow‐up pathways tailored to individual needs offers considerable benefits in terms of patients' experiences following a cancer diagnosis, as well as making services more efficient and cost‐effective.^28^

In order to practise personalised care in an AS setting, strong leadership, and organisational and environmental support^29^ is required to change current HCP thinking and practices towards promoting the need for AS psychosocial support. This additional effort may present challenges within the current health care climate where the exigencies of everyday clinical practice are overwhelming. However, reducing the patients' dependence on HCPs by increasing their sense of control and well‐being through education is a more intelligent, efficient and effective way of working to increase adherence to AS.

### 4.1 Strengths and limitations

Our findings of differences in the ranking of statements by the two panels demonstrate the importance of seeking both the opinions of health care professionals and patient and public engagement to identify priorities for AS psychosocial support. One criticism of the Delphi method is the potential for low feedback rates due to the requirement for multiple rounds of answers, which is integral to the concept of this process.^13^ However, response rates in the current study were high, with only three participants failing to complete the second round of the survey. This may be due in part to the availability and popularity of internet‐based research tools that serve to mitigate Delphi's limitations. The breadth of opinion, numbers of patients and professionals engaged and speed of survey returns confirm the strength of our method. Another criticism of the Delphi technique is its potential to mould opinion.^30^ This is demonstrated in studies that found that participants rate their responses differently when feedback between rounds was distorted.^31^ However, in this Delphi process, feedback provided between rounds was reviewed by the expert ASDG, thereby lessening bias associated with single researcher interpretation. Lastly, patient and carer perspectives were based on feedback from participants who were predominantly from South West London, potentially limiting its generalisability.

## 5 CONCLUSION

This consensus statement was developed to drive national recommendations and local health care improvements to address the support needs of men on AS for low–intermediate risk PCa. Our findings highlight that a structured approach to psychosocial care is desired by both patients and professionals. To increase adherence to AS, it is imperative that patients, HCPs and charities collaborate to co‐design supportive interventions that reflect the views of all key stakeholders involved. This consensus statement is a useful guide for this process.

## CONFLICT OF INTEREST

All authors declare no conflicts of interest.

## AUTHOR CONTRIBUTIONS

NK, MVH, DC, CB and KB performed the concept and design; NK, DC and CB did the data acquisition; NK, KB, DC, CB and MVH did the analysis and interpretation; KB drafted the manuscript; DC, CB, MVH, NK and KB did the critical revision of the manuscript; MVH performed the supervision.

## Supporting information


**Figure S1.** Patient Partner and Family Survey for Delphi process
**Figure S2.** Health care professional Survey for Delphi process
Suppl. Table 1. Patient and Carer Delphi survey participant characteristics
Suppl. Table 2 Healthcare professional Delphi survey participant characteristics
