# Supplementary material for: Developing a consensus statement for psychosocial support in active surveillance for prostate cancer
Source: BJUI Compass. 2022 May 6;4(1):104–13. doi: 10.1002/bco2.155 (PMC9766868; doi:10.1002/bco2.155)
Supplement: Supplementary file 1 — Figure S1. Patient Partner and Family Survey for Delphi process Figure S2. Health care professional Survey for Delphi process Suppl. Table 1. Patient and Carer Delphi survey participant characteristics Suppl. Table 2 Healthcare professional Delphi survey participant characteristics [file BCO2-4-104-s001.docx]

**Supplementary material**

***Figure S1. Patient Partner and Family Survey for Delphi process***

# Active surveillance - Patient, partner and family survey

**1. Introduction**

**Please help us educate healthcare professionals - This survey will take less than 10 minutes of your time.

Dear Participant,
Over 30% of men with prostate cancer are diagnosed with very slow growing cancer where `Active Surveillance' (AS) is recommended. However, a 2016 a Royal College of Surgeons audit suggested that men were over-treated for low risk prostate cancer by as much as 94% in some areas of the UK

Background to our project
Our recently published research identified many factors that influence a man's choice to choose and then remain on active surveillance. The National Institute of Health Research (NIHR) acknowledged this review as evidence to action a change in practice. We would therefore like to understand which of these factors are most helpful or influential. This evidence will help us design an active surveillance clinical service that is fit for the future with appropriate support and information resources.

Who can complete the questionnaire?
We would like to invite men currently on active surveillance, men that have dropped out of active surveillance, partners/family of men on active surveillance or who were on active surveillance to complete this questionnaire.

What do we need you to do?
We need you to complete 2 questionnaires. Once we have reviewed the answers from the first questionnaire we will send the second questionnaire to you directly by email. The second questionnaire will have many of the same questions but will allow us to narrow down and prioritise your answers. The more responses we get, the more accurate and helpful this process will be in focusing NHS resources.

How will you find out about the results of this study?
This questionnaire is part of a PhD project. Once the results have been collected and collated we hope to publish it. We will update you by the end of 2019.

Many thanks in anticipation of your help
Netty Kinsella - Nurse Consultant and PhD Candidate, Royal Marsden Hospital and Kings College London
Mieke Van Hemelrijck - Reader, Kings College London
Christian Brown - Consultant Urologist, King's College Hospital and Guy's and St Thomas' Hospital
Declan Cahill - Consultant Urologist, The Royal Marsden**

**2. Details about Participant**

### **1. What is your Age? ***

|  | Please select age range |
| --- | --- |
| Please select age range | \|  \| \| --- \| |

### **2. How many months have you/your partner/family member been on active surveillance (AS)? ***

|  |
| --- |

### **3. Where do you live (please indicate county (or borough of London) or country if living outside of the UK? ***

|  |
| --- |

### **4. Please select the circle that best describes you: ***

|  | Patient that was previously on active surveillance |
| --- | --- |
|  | Partner |
|  | Other |
|  | Family member |
|  | Patient on active surveillance |

### **5. What is your ethnicity? ***

| • **White** |  |
| --- | --- |
|  | British |
|  | Irish |
|  | Other  • **Asian or Asian British** |
|  | Indian |
|  | Pakistani |
|  | Bangladeshi |
|  | Any other Asian background  • **Mixed** |
|  | White and Black Caribbean |
|  | White and black African |
|  | White and Asian |
|  | Any other mixed background  • **Black or Black British** |
|  | Caribbean |
|  | African |
|  | Any other black background  • **Other Ethnic Group** |
|  | Chinese |
|  | Any other Ethnic Group |
|  | I do not wish to disclose my ethnic origin |

### **6. How long did you spend in education: ***

|  | School |
| --- | --- |
|  | Vocational Qualification/Apprenticeship |
|  | College (up to 18 yrs) |
|  | University Diploma/Degree |
|  | University Higher Degree |

### **7. What is your email address? ***

|  |
| --- |

### **8. Would you like to receive an email updating you on this project?**

|  | Yes |
| --- | --- |
|  | No |
|  | Other (please specify):   \|  \| \| --- \| |

**3. Survey Questions**

### **9. Patient and Family factors: On a scale of 1 (not at all) to 7 (most) how important do YOU think the following factors are whilst on active surveillance. (please click on the circle that best represents how you feel) ***

|  | 1 Not At All | 2 A Little | 3 Some | 4 More | 5 Very Much | 6 Much More | 7 Most |
| --- | --- | --- | --- | --- | --- | --- | --- |
| a. Feeling involved in decisions about active surveillance e.g. scans and re-biopsy |  |  |  |  |  |  |  |
| b. Good physical health whilst on active surveillance |  |  |  |  |  |  |  |
| c. Good mental health whilst on active surveillance |  |  |  |  |  |  |  |
| d. Access to lifestyle advice from a professional (in relation to prostate cancer) |  |  |  |  |  |  |  |
| e. Access to exercise advice from a professional (in relation to prostate cancer) |  |  |  |  |  |  |  |
| f. Access to exercise sessions aimed at men with prostate cancer |  |  |  |  |  |  |  |
| g. Access to dietary advice from a professional (in relation to prostate cancer) |  |  |  |  |  |  |  |
| h. Access to classes in meditation or mindfulness techniques |  |  |  |  |  |  |  |
| i. Including partner/family in consultations and cancer decisions |  |  |  |  |  |  |  |
| j. Partners/family awareness and knowledge of active surveillance |  |  |  |  |  |  |  |
| k. Partners/family acceptance of active surveillance |  |  |  |  |  |  |  |
| l. Access to a local support group |  |  |  |  |  |  |  |
| m. Recommendation from the hospital clinical team |  |  |  |  |  |  |  |
| n. Access to self-management classes |  |  |  |  |  |  |  |
| o. Access to reliable sources reporting on the latest research in prostate cancer and active surveillance |  |  |  |  |  |  |  |

### **10. Cancer factors: On a scale of 1 (not at all) to 7 (most) how important do YOU think the following factors are whilst on active surveillance. (please click on the circle that best represents how you feel) ***

|  | 1 Not at all | 2 A Little | 3 Some | 4 More | 5 Very Much | 6 Much More | 7 Most |
| --- | --- | --- | --- | --- | --- | --- | --- |
| a. Understanding prostate cancer |  |  |  |  |  |  |  |
| b. Understanding the risk of prostate cancer progressing |  |  |  |  |  |  |  |
| c. Understanding the pathology of prostate cancer (Gleason grade) |  |  |  |  |  |  |  |
| d. Understanding the role of PSA in active surveillance |  |  |  |  |  |  |  |
| e. Understanding the role of tumour/cancer volume in active surveillance |  |  |  |  |  |  |  |
| f. Understanding the stage of cancer in relation to active surveillance (T score) |  |  |  |  |  |  |  |
| g. Understanding MRI scans and the role they play in active surveillance |  |  |  |  |  |  |  |
| h. Understanding the other treatment options for prostate cancer |  |  |  |  |  |  |  |
| i. Understanding the side effects of other treatment options for prostate cancer |  |  |  |  |  |  |  |
| j. Control of health - including regular assessment of any prostate related symptoms e.g. urinary symptoms |  |  |  |  |  |  |  |
| k. Hearing/reading stories about public role models on active surveillance for their prostate cancer |  |  |  |  |  |  |  |

### **11. Healthcare provider factors: On a scale of 1 (not at all) to 7 (most) how important do YOU think the following factors are whilst on active surveillance. (please click on the circle that best represents how you feel) ***

|  | 1 Not at all | 2 A Little | 3 Some | 4 More | 5 Very Much | 6 Much More | 7 Most |
| --- | --- | --- | --- | --- | --- | --- | --- |
| a. Easy access to the clinical team |  |  |  |  |  |  |  |
| b. Regular contact with the clinical team (nurse or doctor) via phone |  |  |  |  |  |  |  |
| c. Regular contact with the clinical team (nurse or doctor) via email |  |  |  |  |  |  |  |
| d. The clinical team supporting and recommending active surveillance |  |  |  |  |  |  |  |
| e. Access to reliable information about active surveillance |  |  |  |  |  |  |  |
| f. Access to up to date research on large active surveillance studies |  |  |  |  |  |  |  |
| g. Sharing treatment decision making with the clinical team |  |  |  |  |  |  |  |
| h. Consistently seeing the same clinical team (doctor or nurse) |  |  |  |  |  |  |  |

### **12. Healthcare organisation factors: On a scale of 1 (not at all) to 7(most), how influential do YOU think the following factors are whilst on active surveillance. (please click on the circle that best represents how you feel) ***

|  | 1 Not at all | 2 A Little | 3 Some | 4 More | 5 Very Much | 6 Much More | 7 Most |
| --- | --- | --- | --- | --- | --- | --- | --- |
| a. Agreement on guidelines for safe active surveillance |  |  |  |  |  |  |  |
| b. The hospital cancelling or moving outpatient appointments |  |  |  |  |  |  |  |
| c. The hospital cancelling or moving a biopsy date |  |  |  |  |  |  |  |
| d. The hospital cancelling or moving an MRI scan date |  |  |  |  |  |  |  |
| e. Difficulty contacting the clinical team |  |  |  |  |  |  |  |
| f. Difficulty contacting the administrative team |  |  |  |  |  |  |  |
| g. Being monitored in a cancer centre |  |  |  |  |  |  |  |
| h. Being monitored by a team/clinician with a special interest in active surveillance |  |  |  |  |  |  |  |

### **13. Improving support and Information: On a scale of 1 (not at all) to 7 (most) how do YOU think support and information on active surveillance is best delivered (please click on the circle that best represents how you feel) ***

|  | 1 Not at all | 2 A Little | 3 Some | 4 More | 5 Very Much | 6 Much More | 7 Most |
| --- | --- | --- | --- | --- | --- | --- | --- |
| a. Face to face "information and support seminar" given to a group of men on active surveillance (hospital based) |  |  |  |  |  |  |  |
| b. Face to face 'information and support seminar' given to a group of men on active surveillance (at a local community centre) |  |  |  |  |  |  |  |
| c. Online website (webinar) |  |  |  |  |  |  |  |
| d. A health care professional (doctor, nurse, physio etc) |  |  |  |  |  |  |  |
| e. An expert patient |  |  |  |  |  |  |  |
| f. An online patient forum/chat room |  |  |  |  |  |  |  |
| g. A support group for men on active surveillance |  |  |  |  |  |  |  |
| h. A smartphone app. |  |  |  |  |  |  |  |
| i. Hospital leaflets or booklets given in clinic |  |  |  |  |  |  |  |
| j. Charity sponsored leaflets and booklets |  |  |  |  |  |  |  |
| k. Easy access to community based cancer counsellors |  |  |  |  |  |  |  |

### **14. Delivering follow-up: On a scale of 1 (not at all) to 7 (most) how do YOU think follow-up is best delivered where results of blood tests, scans or biopsies are given (please click on the circle that best represents how you feel). ***

|  | 1 Not at all | 2 A Little | 3 Some | 4 More | 5 Very Much | 6 Much More | 7 Most |
| --- | --- | --- | --- | --- | --- | --- | --- |
| a. Face to face appointments |  |  |  |  |  |  |  |
| b. Telephone appointments |  |  |  |  |  |  |  |
| c. Email from the clinical team |  |  |  |  |  |  |  |
| d. Text message from the clinical team |  |  |  |  |  |  |  |
| e. Skype or FaceTime call from the clinical team |  |  |  |  |  |  |  |
| f. By a GP |  |  |  |  |  |  |  |
| g. By a hospital doctor |  |  |  |  |  |  |  |
| h. By a Specialist Nurse |  |  |  |  |  |  |  |
| i. In a specialist active surveillance clinic |  |  |  |  |  |  |  |

***Figure S2. Health care professional Survey for Delphi process***

# **Active Surveillance - HCP Survey**

**1. Introduction**

**WE NEED YOUR HELP PLEASE - THIS SURVEY WILL TAKE LESS THAN 10 MINUTES TO COMPLETE

Dear Healthcare professional,
Over 30% of men with prostate cancer are diagnosed with low volume, indolent cancer where `Active Surveillance' (AS) is recommended. However, a 2016 a Royal College of Surgeons audit suggested that men were over-treated for low risk prostate cancer by as much as 94% in some areas of the UK

Background to our project
Our 2018 systematic review on active surveillance (published in European Urology) identified many factors that influence a man's choice to both select and remain on active surveillance. The National Institute of Health Research (NIHR) recently acknowledged this review as evidence to action a change in practice. However, we would like to understand which of these factors are most influential. 

What are we trying to achieve?
1. Using the list of factors identified in the review, we are using a`Delphi method'  - 'survey' - to prioritise patients/family supportive care needs whilst on active surveillance. This knowledge will help us design future support and information resources to increase selection and long-term adherence to AS,
2. We would also like to evaluate whether health professionals priorities are aligned with patients 

How will you find out about the results of the questionnaire?
This survey is part of a PhD project. Once the results have been collected and collated we hope to publish it.
We will keep you updated.

Many thanks in anticipation of your help
Netty Kinsella - Nurse Consultant and PhD Candidate, Royal Marsden Hospital and Kings College London
Mieke Van Hemelrijck - Reader, Kings College London
Christian Brown - Consultant Urologist, King's College Hospital and Guy's and St Thomas' Hospital
Declan Cahill - Consultant Urologist, The Royal Marsden**

**2. Details about Participant**

### **1. If you would like to be updated on this project, please provide your Email Address**

|  |
| --- |

### **2. How old are you? ***

|  | Please select age range |
| --- | --- |
| Please select age range | \|  \| \| --- \| |

### **3. What county/country do you work in? ***

|  |
| --- |

### **4. Please select the circle that best describes you: ***

|  | Hospital Consultant |
| --- | --- |
|  | Hospital Trainee |
|  | GP |
|  | GP Trainee |
|  | Nurse |
|  | Associate Health Professional |

**3. Survey Questions**

### **5. Patient and Family Factors The following issues have been identified by patients as 'Patient and Family Factors' that effect their adherence to Active surveillance. On a scale of 1 (not at all) to 7 (most) how important do YOU think the following factors are whilst on active surveillance. (please click on the circle that best represents how you feel) ***

|  | 1 Not At All | 2 A Little | 3 Some | 4 More | 5 Very Much | 6 Much More | 7 Most |
| --- | --- | --- | --- | --- | --- | --- | --- |
| a. The patient feeling involved in decisions about monitoring active surveillance |  |  |  |  |  |  |  |
| b. Quality of physical health whilst on active surveillance |  |  |  |  |  |  |  |
| c. Quality of mental health whilst on active surveillance |  |  |  |  |  |  |  |
| d. Access to lifestyle advice from a professional (in relation to prostate cancer) |  |  |  |  |  |  |  |
| e. Access to exercise advice from a professional (in relation to prostate cancer) |  |  |  |  |  |  |  |
| f. Access to exercise sessions aimed at men with prostate cancer |  |  |  |  |  |  |  |
| g. Access to dietary advice from a professional (in relation to prostate cancer) |  |  |  |  |  |  |  |
| h. Access to classes in meditation or mindfulness techniques |  |  |  |  |  |  |  |
| i. Including partner/family in consultations and cancer decisions |  |  |  |  |  |  |  |
| j. Partners/family awareness and knowledge of active surveillance |  |  |  |  |  |  |  |
| k. Partners/family acceptance of active surveillance |  |  |  |  |  |  |  |
| l. Access to a local support group |  |  |  |  |  |  |  |
| m. Recommendation from the hospital clinical team |  |  |  |  |  |  |  |
| n. Access to self-management classes |  |  |  |  |  |  |  |
| o. Access to reliable sources reporting on the latest research in prostate cancer and active surveillance |  |  |  |  |  |  |  |

### **6. Cancer Factors The following issues have been identified by patients as 'Cancer factors' that effect their adherence to Active surveillance On a scale of 1 (not at all) to 7 (most) how important do YOU think the following factors are whilst on active surveillance. (please click on the circle that best represents how you feel) ***

|  | 1 Not at all | 2 A Little | 3 Some | 4 More | 5 Very Much | 6 Much More | 7 Most |
| --- | --- | --- | --- | --- | --- | --- | --- |
| a. Understanding prostate cancer |  |  |  |  |  |  |  |
| b. Understanding the risk of prostate cancer progressing |  |  |  |  |  |  |  |
| c. Understanding the pathology of prostate cancer (Gleason grade) |  |  |  |  |  |  |  |
| d. Understanding the role of PSA in active surveillance |  |  |  |  |  |  |  |
| e. Understanding the role of tumour volume in active surveillance |  |  |  |  |  |  |  |
| f. Understanding the stage of cancer in relation to active surveillance (T scores) |  |  |  |  |  |  |  |
| g. Understanding MRI scans and the role they play in active surveillance |  |  |  |  |  |  |  |
| h. Understanding the other treatment options for low-intermediate risk prostate cancer |  |  |  |  |  |  |  |
| i. Understanding the side effects of other treatment options for prostate cancer |  |  |  |  |  |  |  |
| j. Control of health - including regular assessment of any prostate related symptoms e.g. urinary symptoms |  |  |  |  |  |  |  |
| k. Hearing/reading stories about public role models on active surveillance for their prostate cancer |  |  |  |  |  |  |  |

### **7. Healthcare provider factors: The following issues have been identified by patients as 'Healthcare provider factors' that effect their adherence to Active surveillance On a scale of 1 (not at all) to 7(most) how important do YOU think the following factors are whilst on active surveillance. (please click on the circle that best represents how you feel) ***

|  | 1 Not at all | 2 A Little | 3 Some | 4 More | 5 Very Much | 6 Much More | 7 Most |
| --- | --- | --- | --- | --- | --- | --- | --- |
| a. Easy access to the clinical team |  |  |  |  |  |  |  |
| b. Regular contact with the clinical team (nurse or doctor) via phone |  |  |  |  |  |  |  |
| c. Regular contact with the clinical team (nurse or doctor) via email |  |  |  |  |  |  |  |
| d. The clinical team supporting and recommending active surveillance |  |  |  |  |  |  |  |
| e. Access to reliable information about active surveillance |  |  |  |  |  |  |  |
| f. Access to up to date research on large active surveillance studies |  |  |  |  |  |  |  |
| g. Sharing treatment decision making with the clinical team |  |  |  |  |  |  |  |
| h. Consistently seeing the same clinical team (doctor or nurse) |  |  |  |  |  |  |  |

### **8. Healthcare organisation factors: ​The following issues have been identified by patients as 'Healthcare organisation factors' that effect their adherence to Active surveillance On a scale of 1 (not at all) to 7(most), how influential do YOU think the following factors are whilst on active surveillance. (please click on the circle that best represents how you feel) ***

|  | 1 Not at all | 2 A Little | 3 Some | 4 More | 5 Very Much | 6 Much More | 7 Most |
| --- | --- | --- | --- | --- | --- | --- | --- |
| a. Clear National or Local guidelines for safe active surveillance |  |  |  |  |  |  |  |
| b. The hospital cancelling or moving outpatient appointments |  |  |  |  |  |  |  |
| c. The hospital cancelling or moving a biopsy date |  |  |  |  |  |  |  |
| d. The hospital cancelling or moving an MRI scan date |  |  |  |  |  |  |  |
| e. Difficulty contacting the clinical team |  |  |  |  |  |  |  |
| f. Difficulty contacting the administrative team |  |  |  |  |  |  |  |
| g. Being monitored in a cancer centre |  |  |  |  |  |  |  |
| h. Being monitored by a team/clinician with a special interest in active surveillance |  |  |  |  |  |  |  |

### **9. Support and Information: ​The following suggestions have been made by patients and clinicians to better 'Support and inform' patients on Active surveillance On a scale of 1 (not at all) to 7 (most) how do YOU think support and information on active surveillance is best delivered (please click on the circle that best represents how you feel) ***

|  | 1 Not at all | 2 A Little | 3 Some | 4 More | 5 Very Much | 6 Much More | 7 Most |
| --- | --- | --- | --- | --- | --- | --- | --- |
| a. Face to face "information and support seminar" given to a group of men on active surveillance (hospital based) |  |  |  |  |  |  |  |
| b. Face to face 'information and support seminar' given to a group of men on active surveillance (at a local community centre) |  |  |  |  |  |  |  |
| c. Online website (webinar) |  |  |  |  |  |  |  |
| d. A health care professional (doctor, nurse, physio etc) |  |  |  |  |  |  |  |
| e. An expert patient |  |  |  |  |  |  |  |
| f. An online patient forum/chat room |  |  |  |  |  |  |  |
| g. A support group for men on Active Surveillance |  |  |  |  |  |  |  |
| h. A smartphone app. |  |  |  |  |  |  |  |
| i. Hospital leaflets or booklets given in clinic |  |  |  |  |  |  |  |
| j. Charity sponsored leaflets and booklets |  |  |  |  |  |  |  |
| k. Easy access to community based cancer counsellors |  |  |  |  |  |  |  |

### **10. Delivering follow-up: ​The following suggestions have been made by patients and clinicians to better support 'follow-up' in patients on Active surveillance. On a scale of 1 (not at all) to 7 (most) how do YOU think follow-up is best delivered where results of blood tests, scans or biopsies need to be communicated (please click on the circle that best represents how you feel). ***

|  | 1 Not at all | 2 A Little | 3 Some | 4 More | 5 Very Much | 6 Much More | 7 Most |
| --- | --- | --- | --- | --- | --- | --- | --- |
| a. Face to face appointments |  |  |  |  |  |  |  |
| b. Telephone appointments |  |  |  |  |  |  |  |
| c. Email from the clinical team |  |  |  |  |  |  |  |
| d. Text message from the clinical team |  |  |  |  |  |  |  |
| e. Skype or FaceTime call from the clinical team |  |  |  |  |  |  |  |
| f. By a GP |  |  |  |  |  |  |  |
| g. By a hospital doctor |  |  |  |  |  |  |  |
| h. By a Specialist Nurse |  |  |  |  |  |  |  |
| i. In a specialist active surveillance clinic |  |  |  |  |  |  |  |

**Suppl. Table 1. Patient and Carer Delphi survey participant characteristics**

| Age Range | No. of participants |
| --- | --- |
| 0-19 | 0 |
| 20-29 | 1 |
| 30-39 | 0 |
| 40-49 | 5 |
| 50-59 | 9 |
| 60-69 | 22 |
| 70-79 | 17 |
| 80+ | 1 |
| Ethnicity |  |
| White other | 1 |
| White British | 45 |
| White Irish | 3 |
| Asian | 3 |
| Black African/Caribbean | 3 |
| Level of education |  |
| School | 5 |
| Vocational qualification | 2 |
| College | 9 |
| University Diploma/Degree | 19 |
| University Higher Degree | 20 |

**Suppl. Table 2 Healthcare professional Delphi survey participant characteristics**

| Age Range | No. of participants |
| --- | --- |
| 20-29 | 1 |
| 30-39 | 40 |
|  |  |
| 40-49 | 40 |
| 50-59 | 19 |
| 60-69 | 12 |
| Profession |  |
| Consultant | 65 |
| Hospital Trainee | 25 |
| GP | 0 |
| GP Trainee | 0 |
| Nurse | 23 |
| AHP | 1 |
| Practice area |  |
| Midlands | 2 |
| North of England | 10 |
| South of England | 59 |
| Scotland | 2 |
| Wales | 3 |
| Europe | 10 |
| Rest of the World | 5 |
| Anonymous | 23 |
